# Interplay between Attenuation- and Virulence-Factors of *Babesia bovis* and Their Contribution to the Establishment of Persistent Infections in Cattle

**DOI:** 10.3390/pathogens8030097

**Published:** 2019-07-04

**Authors:** Gina M. Gallego-Lopez, Brian M. Cooke, Carlos E. Suarez

**Affiliations:** 1 Department of Medical Microbiology and Immunology, University of Wisconsin–Madison, Madison, WI 53706, USA; 2 Department of Microbiology, Biomedicine Discovery Institute, Monash University, Melbourne, VIC 3800, Australia; 3 Australian Institute of Tropical Health and Medicine, James Cook University, Cairns, QLD 4878, Australia; 4 Department of Veterinary Microbiology and Pathology, College of Veterinary Medicine, Washington State University, Pullman, WA 99164-7040, USA; 5 Animal Disease Research Unit., Agricultural Research Service, USDA, Pullman, WA 99164-6630, USA

**Keywords:** persistence, Babesia, cytoadhesion, sequestration, VESA, SBP2t11

## Abstract

Bovine babesiosis is an acute and persistent tick-borne global disease caused mainly by the intraerythrocytic apicomplexan parasites *Babesia bovis* and *B. bigemina*. *B. bovis* infected erythrocytes sequester in blood capillaries of the host (cytoadhesion), causing malaria-like neurological signs. Cytoadhesion and antigenic variation in *B. bovis* are linked to the expression of members of the Variant Erythrocyte Surface Antigen (VESA) gene family. Animals that survive acute *B. bovis* infection and those vaccinated with attenuated strains remain persistently infected, suggesting that *B. bovis* parasites use immune escape mechanisms. However, attenuated *B. bovis* parasites do not cause neurological signs in vaccinated animals, indicating that virulence or attenuation factors play roles in modulating parasite virulence phenotypes. Artificial overexpression of the SBP2t11 protein, a defined attenuation factor, was associated with reduced cytoadhesion, suggesting a role for this protein as a key modulator of virulence in the parasite. Hereby, we propose a model that might be functional in the modulation of *B. bovis* virulence and persistence that relies on the interplay among SBP2t, VESA proteins, cytoadhesion, and the immune responses of the host. Elucidation of mechanisms used by the parasite to establish persistent infection will likely contribute to the design of new methods for the control of bovine babesiosis.

## 1. Introduction

**Figure 1:**
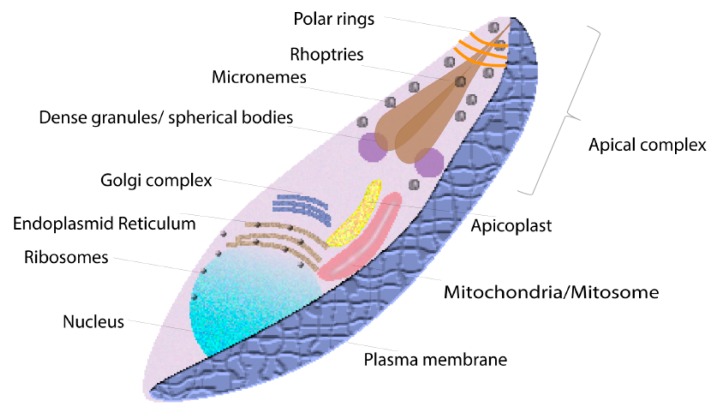
General Apicomplexa structure. Apicomplexan parasites, in addition to nucleus, ribosomes, endoplasmic reticulum, mitochondria, and Golgi complex, have a characteristic apical complex that includes the secretory organelles known as micronemes, rhoptries, dense granules or spherical bodies, conoid, and polar rings. Apicomplexan parasites have an apicoplast (*Cryptosporidium* does not have apicoplast). *Cryptosporidium* have mitochondria reduced in size and function known as a mitosome.

Apicomplexa is a phylum of obligatory parasitic protozoans that are comprised of the obligate endoparasites of animals and humans, which share complex apical structures in their zoite stages and are specialized for cell attachment and invasion [1]. Apicomplexans are among the most successful parasitic organisms existing in nature due to their ability to invade and survive inside different hosts [2]. Although the structure of the apical complex varies slightly among apicomplexans, typically it includes secretory organelles known as micronemes, rhoptries, and dense granules or spherical bodies (Figure 1). Other structures associated with the apical complex are the conoid (only present in coccidian) and polar rings [1]. Additionally, apicomplexan parasites have a non-photosynthetic plastid structure (apicoplast), which probably originated from a red alga by a process of endosymbiosis [3]. The complement of secretory organelles contains proteins needed for cell invasion, a critical parasite function that usually causes pathological consequences for the host, and thus at least some apical complex molecules could be considered as virulence factors. *Babesia* are tick-borne apicomplexan parasites that are the causative agents of babesiosis: one of the most common arthropod-borne infections of free-living animals worldwide [4]. More than 100 species of *Babesia* can infect numerous tick vectors and mammalian hosts, and among them, *Babesia bovis* and *B. bigemina* (the causative agents of bovine babesiosis) have a large impact on the beef and dairy industries in many tropical and semi-tropical regions worldwide. The incidence of bovine babesiosis is increasing due to the lack of available effective vaccines together with an expansion of the geographical range of its tick (*Riphicephalus* spp.) transmission vector (mainly *R. microplus*), largely driven by climatic change and other human interventions. Other important risk factors include increased presence of acaricide-resistant tick vectors, and, in the case of the USA, the increase in cattle importation from endemic countries [5]. An additional source of concern is the risk of transmission resulting from free-moving wild animals that are infested with *Babesia*-infected ticks [6]. Since babesiosis is geographically restricted to tropical and subtropical areas due to environmental requirements by their tick vectors, climate change results in the expansion of the geographical niche of the tick vectors able to transmit the disease into regions that were previously considered as non-endemic in recent years [7]. Overall, babesiosis has a large and significant economic impact on the beef and dairy industries due to mortality, ill-thrift, abortions, loss of milk and meat production, treatment cost, and ultimately, impact on international cattle trade [6].

## 2. Babesia Life Cycle

**Figure 2:**
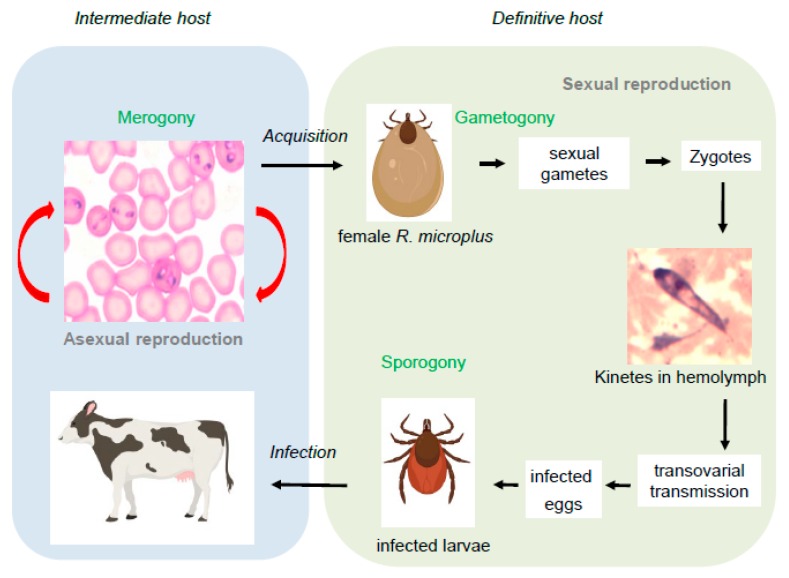
*Babesia bovis* life cycle. It consists of merogony, gametogony, and sporogony. A female *Riphicephalus microplus* feeds on an infected host. The sexual reproduction occurs in the definitive host, the tick. Some of the merozoites transform into sexually reproductive cells, which join in pairs and form gametes (gametogenesis). Pairs of gametes then fuse to form zygotes. The zygote differentiates in a motile kinete, which travel through the hemolymph. Kinetes invade different tick organs including the ovary, so when the tick lay eggs, these eggs are already infected. These eggs developed in infected larvae, where new sporozoites are generated and stored in the salivary glands (sporogony). The infected larvae infect the mammalian host (intermediate host), invading the erythrocytes. Intraerythrocytic, the parasite undergoes asexual reproduction (merogony) and the cycle begins again.

The life cycle of *Babesia* spp. consists of merogony, gametogony, and sporogony (Figure 2). *Babesia* is naturally transmitted transovarially by the bite of infected *Ixodid* tick larva and nymphs for *B. bovis* and *B. bigemina*, respectively. Motile sporozoites stored within the salivary glands of the ticks are injected into the mammalian host, where they infect erythrocytes [8]. Once inside the erythrocyte, sporozoites develop into trophozoites where binary fission occurs forming merozoites. Merozoites egress from infected erythrocytes and re-infect other erythrocytes. Some trophozoites undergo gametogenesis in the midgut of the tick and develop into Strahlenkörper forms. These fuse to form zygotes, which then develop into motile kinetes. Through the hemolymph of the tick, motile kinetes invade different tick organs, including the ovary, where they can access tick eggs, thus leading to transovarial transmission. Finally, the parasites reach the salivary glands of the larva of the next generation of ticks, where the parasites form multi-nucleated sporoblasts. Resulting sporozoites are then injected into the mammalian host as the ticks acquire a blood meal [9]. Inoculation of *Babesia* sporozoites by larva (*B. bovis*) or nymph (*B. bigemina*) stages of the vector *Riphicephalus* ticks into naïve animals may result in acute babesiosis.

## 3. Acute and Persistent Babesia Infections

Acute symptoms of babesiosis are broad, ranging from a sub-clinical infection to a fulminant malaria-like disease, which can be fatal [4]. The severity of the disease depends on the age of the mammalian host, the competency of the host immune system, and co-infection with other pathogens [4]. The only method available to prevent acute bovine babesiosis, at least to some degree, is the use of live-attenuated vaccines. Such vaccines comprise attenuated parasite strains that are derived from virulent strains that have undergone a series of rapid passages in calves [10,11,12]. The *B. bovis* attenuated vaccine strains are generated by rapid serial passages (usually 22 to 28) of blood containing infected erythrocytes in splenectomized calves [11,13], while *B. bigemina* vaccine strains are generated by serial passages of infected erythrocytes in spleen intact calves [14]. Again, the attenuated parasites cause mild disease in vaccinated animals, but these parasites also can transit into persistent disease, on the face of the immune response of the hosts. The mechanisms involved in the attenuation of *Babesia* parasites by passage in calves remain unknown. However, among other possibilities, attenuation may be due to the selection of a preexisting attenuated subpopulation in the virulent strain by the successive passages, or due to the occurrence of genetic or epigenetic changes in the parasites resulting in changes of their virulence phenotype. Regardless of the mechanisms involved, comparisons of virulent/attenuated, genetically-related pairs of parasites using genomic and transcriptomic approaches allowed the discovery of genes that are differentially expressed in attenuated parasites, as described below.

Although highly genetically related, *B. bovis* and *B. bigemina* cause distinct pathologies in infected cattle. While both parasites are responsible for increased fever and anemia, and for other signs related to massive destruction of erythrocytes, *B. bigemina* causes milder acute disease followed by persistent disease in surviving animals. In contrast, virulent strains of *B. bovis* cause acute infections, characterized by neurological signs such as ataxia, lethargy, and inappetence [15]. Neurovirulence is most likely related to the cytoadhesion of infected erythrocytes to vascular endothelial cells and their subsequent sequestration in the cerebral microvasculature [16,17], resulting in altered blood flow and inflammation [18]. Neither sequestration nor neurological signs occur in animals infected with *B. bigemina*. However, the receptors involved in the interactions between *B. bovis* infected erythrocytes and host endothelial cells remain unknown. It is possible that changes in the surface architecture of the infected erythrocytes, such as ridges mediated by the parasites, facilitate this process. In addition, parasite proteins expressed on the surface of the infected erythrocyte, such as the members of the Variant Erythrocyte Surface Antigen (VESA) family, have been identified as possible candidates for mediating such interactions, but it is possible that other still uncharacterized molecules participate directly or indirectly in this mechanism. Less is known about molecules expressed on the endothelial cells lining the vasculature that can bind to receptors expressed on the surface of infected erythrocytes. Animals infected with virulent strains of *Babesia* parasites also suffer from hyperthermia (≥39 °C) for three or more days, with decreases in hematocrit greater than 40%. These, combined severe clinical signs, usually require chemotherapeutic intervention to prevent the fast demise of infected animals. In contrast, *B. bovis* attenuated strains derived artificially from the virulent parental strains do not induce neurovirulence in inoculated young calves, which are usually more resistant to infection [17,18]. Additionally, hosts infected with attenuated strains display reduced erythrocyte sequestration, less hyperthermia, and a lower decrease in hematocrit [17,18]. These vaccinated animals survive with no chemotherapeutic intervention, and thus live vaccines based on attenuated *B. bovis* strains are frequently used in endemic countries. Cytoadhesion of infected erythrocytes remains an important mechanism that enables the development of persistent infection, which is subsequent to the acute stage. A feature of persistent infection is low levels of fluctuating parasitemia, which results in the occurrence of asymptomatic cattle in endemic areas that function as reservoirs that assure transmission of the parasite. 

Immunological studies have demonstrated that an important mechanism that protects cattle from severe manifestations of the disease is the clearance of the parasites from the circulation mediated by macrophages in the spleen [19,20,21]. However, there is consensus that the ability of *B. bovis* to sequester in capillaries of the host (mainly in the brain and kidney, as well as in other organs) helps the parasite to avoid the immune responses of the hosts, and particularly, the trapping of infected erythrocytes mediated by macrophages residing in the spleen of the hosts. This ‘parasite escape’ strategy is similar to the mechanisms used by *Plasmodium* parasites to avoid clearance by the host immune system [22,23]. Furthermore, and in a fashion similar to *Plasmodium*, sequestration of infected erythrocytes in brain microvasculature is also responsible for the generation of the neurological symptoms [17,24]. The mechanisms involved in sequestration of *B. bovis* remain poorly defined, but it is known that *B. bovis* express proteins that can produce important modifications in the architecture of the infected erythrocyte [25,26,27,28,29]. In addition to cytoadhesion and sequestration, the parasite is also able to generate antigenic diversity in the molecules exposed on the erythrocyte surface, such the members of the VESA family, to escape the antibody responses of the host [30,31]. In summary, cytoadhesion and rapid antigenic variation acting together are widely considered as the main mechanisms used by *B. bovis* to allow persistent infections.

## 4. Immune Mechanisms of Persistent *Babesia* Infections

The outcome of a *B. bovis* infection depends on the balance between host and parasite responses to the inflammatory response induced by the parasite. After acute infection, animals either die or recover from the infection either naturally or after drug treatment. After recovery, the parasite establishes persistency and the host become chronically infected [19].

**Figure 3:**
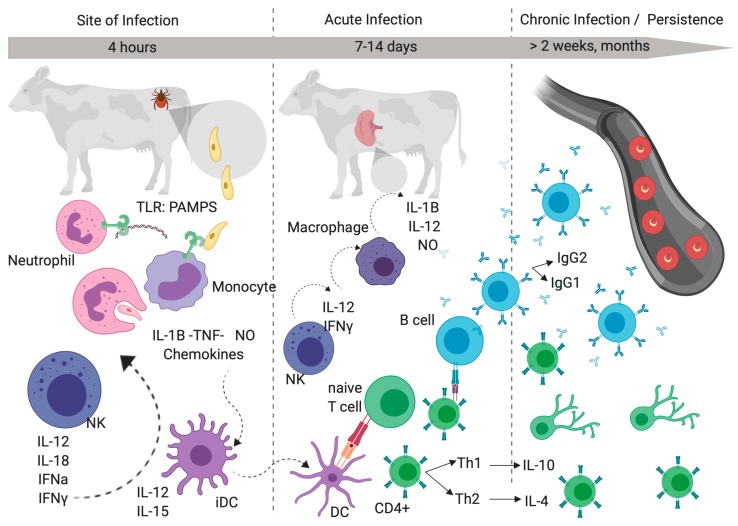
Immune regulation of *Babesia* infection toward Persistence. During the bite, the ticks insert their mouthparts into the skin on their host and cause tissue injury, creating hemorrhagic pools where *Babesia* sporozoites stimulate host innate immune response. *Babesia* pathogens associated molecules patterns (PAMPS) bind to the mammalian toll-like receptors (TLRs) on neutrophils or monocytes. Monocytes or phagocytic neutrophils release cytokines such as IL-B, TNF-α, and Nitric Oxide (NO), and chemokines attract immature dendritic cells (iDC). Natural Killer cells (NK) release interleukines (IL) such as IL-12, IL-18, IFNα, and IFNγ. iDC release IL-12 and IL-15. Mature DC (mDC) migrate to secondary lymphoid tissue such as the spleen or lymph nodes. In the spleen, the mDC present *Babesia* antigens to naïve T cells. Spleen macrophages activated by IFNγ kill the parasite by phagocytosis, and kill the activity of toxic metabolites such as NO and secreted products such as 1L-1β, 1L-12, and TNF-α, which inhibit *B. bovis* growth. The mature DC present antigens to CD4+ T cells. CD4+ T cells produce IFN-γ and IL-4. B cells produce IgG1 and opsonizing IgG2 antibodies. Control of acute *Babesia* infection results in the development of chronic disease and persistence where adaptive immune response is present. During persistent *B. bovis* infection, there are not evident clinical symptoms, but the animals still have low fluctuating parasitemia because infected erythrocytes cytoadhered to the host microvasculature.

Following a tick bite, at the site of infection, an innate immune response against infected erythrocytes or free *Babesia* merozoites is initiated [8]. Different *Babesia* pathogen associated molecular patterns (PAMPs) bind to mammalian toll-like receptors (TLRs) expressed on neutrophils or monocytes, a process responsible for the initiation of the host innate immune response. Mainly, non-methylated CpG motifs of DNA bind to bovine TLR9 [32,33]. Monocytes or phagocytic neutrophils release cytokines such as IL-B, TNF-α, and Nitric Oxide (NO) in *Babesia* infections [34] and in chemokines that attract immature dendritic cells (iDC). Also, Natural Killer cells (NK) have been identified in the early stages of *Babesia* infection [35]. NKs release interleukin (IL) IL-12, IL-18, and IFNγ [21,36]. Mature DC (mDC) migrate to secondary lymphoid tissue, such as the spleen or lymph nodes, at the beginning of the acute infection, which is characterized by high parasitemia, fever, anemia, drop of hematocrit, and organ failure. In the spleen, the mDC present *Babesia* antigens to naïve T cells. Spleen macrophages activated by IFNγ [21,36] kill the parasite by phagocytosis and the activity of toxic metabolites such as NO [34]. The important role of macrophage activation was demonstrated by in vitro experiments where macrophages secreted products such as 1L-1β, 1L-12, and TNF-α, which inhibited in vitro *B. bovis* growth [21] (Figure 3). 

**Figure 4:**
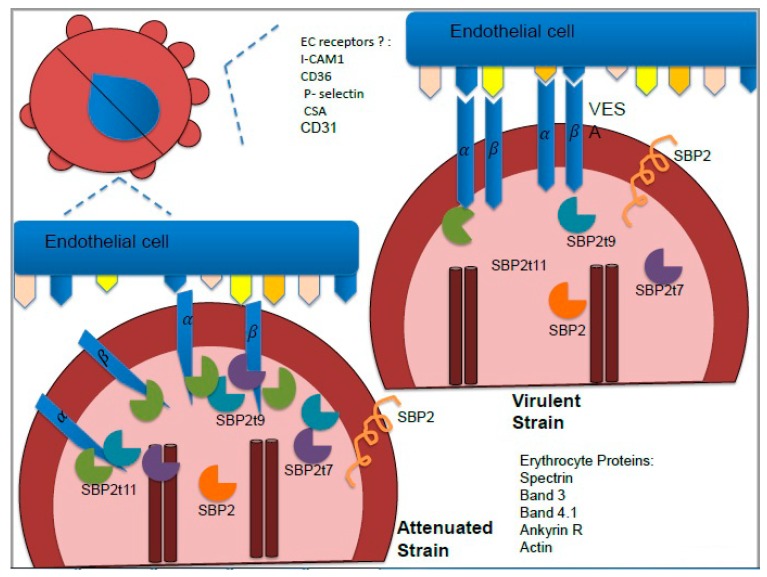
Proposed model of a possible mechanism of attenuation in *Babesia bovis*. SBP2 and SBP2 truncated proteins (SBP2t7, SBP2t9, and SBP2t11) might be involved in protein-protein interactions, in a mode that inhibits or regulates ridge formation in *B. bovis* infected erythrocytes. In the virulent *B. bovis* infected-erythrocytes (right figure), *sbp2* truncated transcripts are down-regulated, which could correlate to their protein levels; Variant Erythrocyte Surface Antigen (VESA) proteins forming the heterodimer VESAαβ are expressed on the surface on infected erythrocytes, especially in the tips of numerous ridges. Thus, the VESA heterodimer is interacting with undefined receptors on the endothelial cells. These receptors could be EC receptors, I-CAM1, CD36, P-selectin, CSA, or CD31 as it occurs in *Plasmodium.* Thus, cytoadhesion in a virulent strain to endothelial cells is highly abundant. On the left side, in the attenuated *B. bovis* strain infected erythrocyte, *sbp2* truncated transcripts are up-regulated, which could correlate to their protein levels; VESA proteins are not forming the heterodimer VESAαβ, and the number of ridges is reduced. Thus, the binding to VESA-receptors is interrupted and cytoadhesion to endothelial cells is reduced. Additionally, undefined erythrocyte cytoskeleton proteins (Spectrin, Band 3, Band 4.1, Ankyrin R, and Actin) could be interacting with SBP proteins as well.

Acquired immunity after immunization with a *B. bovis* vaccine or recovery from infection is based on CD4+ T cells, macrophages, and antibody production [20]. The mature DC present antigens to CD4+ T cells. CD4+ T cells levels were high from animals protected against *B. bovis* infection [37]. Activated spleen macrophages produce cytokines, such as IFN-α, IL-12, and IL-18, that stimulate CD4+ T cells. CD4+ T cells produce IFN-γ and IL-4 [38]. IFN-γ is required for activating macrophages to produce babesiacidal molecules and for enhancing an opsonizing IgG2 antibody response. IL-4 produced by CD4+ enhances IgG1 production. Both antibody and activated CD4+ T cells are important for maintaining protective immunity in *B. bovis* [20] (Figure 4). However, because *Babesia* parasites reside inside erythrocytes, which do not express MHC molecules, mechanisms involving direct effector activities of lymphocytes, such as cytotoxic lymphocytes, are not considered relevant for effective control. 

The outcome of the acute infection depends on the timing, production, and quantity of cytokine response. Innate response is protective when IFNγ and IL-12 are produced before IL-10 production. However, the innate response is not protective when IL-10, IL-12, and IFNγ are produced at the same time [39]. Control of acute *Babesia* infection results in the development of chronic disease and persistence where adaptive immune response is present. During persistent *B. bovis* infections, there are not evident clinical symptoms because the infection is controlled, but the animals still have low fluctuating parasitemia. It is possible that parasite persistence, in the face of a strong immune response, is aided by the cytoadhesion of infected erythrocytes to the host microvasculature, which allows the parasite to avoid elimination by spleen macrophages. It has been suggested that it is beneficial for the parasite to induce sufficient protective immunity that would prevent death of the host, facilitate parasite persistence, and assure further transmission to a new host [19] (Figure 3). Thus, persistence results in a balance where the parasite assures its transmission to the host and its survival. It is also possible that the parasite puts into action mechanisms that facilitate persistence in an unfavorable environment where it can co-exist with the strong defenses mediated by the immune system of the host, such as antigenic variation and sequestration, and/or by modulating its virulence. In addition, *Babesia* parasites can also use mechanisms of persistence based on the modulation or interference with the effector arms of the immune system, as exemplified by the expression of immunoglobulin receptors on the surface of infected erythrocytes by *B. bigemina* [40]. 

## 5. Interplay among Babesia and Its Hosts

*Babesia* parasites have co-evolved with their definitive arthropod tick vectors and the intermediate vertebrate hosts developing mechanisms that lead to parasite perpetuation. These mechanisms involve the production and interplay of virulence and attenuation factors. However, the interactions among the parasite and the vertebrate host are also strongly influenced by the responses of the immune system. The immune system of the bovine is known to generate a massive humoral immune response against the detection of bovine babesiosis [41,42,43], upon controlling acute infections. The result of these interactions is usually a balance that is mainly reached upon the establishment of persistent infections where the parasite assures its transmission and host survival. 

The restriction on the expansion of parasite numbers in persistently infected animals occurs despite the expression of variable VESA antigens (see below) on the surface of the infected erythrocytes, which is regarded as an escape strategy of the parasite. This observation suggests that effective control of the number of circulating parasites could be due mainly to the destruction of free merozoites, rather than infected erythrocytes. This is supported by the large amount of surface merozoites antigens recognized by sera of persistently infected cattle, including antigens of the Variable Merozoite Surface Antigen family (VMSA) [44] and the absence of clinical manifestations, such as splenomegaly and signs of hemolytic anemia, in persistently infected animals. The VMSA is a family of antigens, that includes MSA-1 and MSA2a, b, and c, that is not subjected to fast antigenic variation, but it is in contrast highly variable among distinct strains of the parasite [45,46,47]. Overall, these observations suggest the existence of a unique interplay between the parasite and the immune system of the host where both the host and the parasites are benefited. 

On the other hand, cytoadhesion thus prevents infected erythrocytes from circulating through the spleen where they would be eliminated by phagocytosis. This mechanism seems to be operative during both acute and persistent infections and is central to control the infection. Paradoxically, parasite survival relies on the occurrence of an effective immune system, which can regulate uncontrolled development of the parasite in the host for its survival at the population level, and thus expresses attenuation factors that can modulate the virulence and move the tip in the direction of survival for both the host and the parasite.

## 6. Possible Role of Attenuation Factors in Modulating Persistent Infection and Virulence

The mechanism underlying parasite attenuation through passage is not fully understood, but some evidence of changes occurring at the genomic, transcriptomic, proteomic, and pathophysiological levels has been collected so far. First, comparative genomic analysis of *B. bovis* virulent and attenuated strain pairs suggested minimal changes at the coding level among the virulent and attenuated strain pairs, and attenuation may be a strain-specific acquired phenotype, although all attenuated derivatives consist of significantly reduced genomic content [48].

Second, the subsequent transcriptome analysis of two virulent and attenuated strain pairs revealed significant differential gene expression. Specifically, three spherical body protein (SBP) 2 gene family members were found up-regulated in the Argentina and Texas attenuated strains: *sbp2t7*, *sbp2t9,* and *sbp2t11* [49]. Additionally, *sbp2t11* was found consistently up-regulated in multiple attenuated strains, when compared to their virulent counterparts [50]. This altered gene expression pattern, exclusive to the attenuated strains, suggests that *sbp2t11* may be a universal attenuation marker for *B. bovis* [49,50]. 

Third, there is likely a connection between low cytoadhesion and attenuation [34]. As it was mentioned previously, cytoadhesion in bovine babesiosis occurs as *B. bovis* infected erythrocytes adhere to capillary endothelial cells in vivo, resulting in the concentration of infected erythrocytes in the host microvasculature [16,51]. This parasite strategy has been proposed to result in the avoidance of parasite splenic clearance while it facilitates establishing persistent infection [52]. The observation that significant differences in the number of sequestered parasites in cerebral capillaries were detected between animals infected with Texas virulent and attenuated strains [17] suggests that cytoadherence is a mechanism that might be closely correlated with virulence. Consistent with this postulate, artificial up-regulation of the attenuation marker *sbp2t11* was also associated with low cytoadhesion [53].

Fourth, there remains an unexplored relationship between VESA and attenuation. The Variant Erythrocyte Surface Antigen 1 (VESA) protein is the largest protein family in *B. bovis* and it has been associated to cytoadherence and antigenic variation [29,54]. VESA protein is a heterodimer formed by a 1α and a 1β subunit [55]. *Ves* genes encode the VESA proteins; the VESA1α subunit is encoded by >150 *Ves* α genes and the β subunit is encoded by >80 *ves* β genes [56]. *Ves* α and *ves* β are divergently oriented and hypothesized to be transcribed at the locus of active transcription, resulting in the expression of VESA1αβ heterodimer on the erythrocyte surface [54,55,57]. Previously, it was demonstrated that antibodies generated during acute babesiosis infection disrupted infected erythrocytes and endothelial cells bonds [58]. Also, a variable, polymorphic doublet VESA1 protein was immunoprecipitated from this serum using *Babesia* hyperimmune IgG serum to precipitate L-35S methionine-labeled parasite proteins [58]. In addition, VESA proteins were found to be associated with ridges that are present in the surface of *B. bovis* infected erythrocytes. Such ridges are also likely to be responsible for facilitating the cytoadherence of infected erythrocytes to vascular endothelial cells [58], and certain attenuated *B. bovis* strains display reduced numbers of ridges [59,60]. Collectively, these data suggest the possibility that cytoadherence is associated with the expression of VESA proteins [58]. In addition, since VESA proteins are known for their high degree of variation and differential patterns of expression through the infection, it has been suggested that they may also assist the parasite to escape the host immune system. According to a transcriptome analysis [49], in virulent *B. bovis* strains, the variant erythrocyte surface antigen (*ves*) αβ pair genes are up-regulated and divergently oriented meanwhile *sbp* genes are down-regulated. On the other hand, in attenuated *B. bovis* strains, *ves* genes are up-regulated but the heterodimers are unpaired, meanwhile *sbp* genes are up-regulated [49].

The actual molecular mechanisms explaining the relationships among *sbp2t11* levels and cytoadherence remain unknown. VESA proteins and/or other erythrocyte cytoskeleton proteins could be interacting with *Babesia* proteins such as the SBP proteins in a similar panorama as previously reported for *Plasmodium* [61,62,63]. *P. falciparum* erythrocyte membrane protein (PfEMP1) mediates cytoadhesion in *P. falciparum*, undergoes antigenic variation, and is encoded by the *var* multigene family. Thus, the PfEMP1 pattern of expression influences the severity of the malaria disease [64]. Both the N-terminal and the C-terminal portion of PfEMP1 participate in important interactions: the extracellular N-terminal portion of PfEMP1 interacts with different host receptors such as I-CAM1, CD36, P-selectin, and chondroitin sulfate A (CSA); meanwhile, the intracellular C-terminal portion of PfEMP1 interacts with the knob-associated histidine-rich protein (KAHRP), which has been implicated in the formation of knobs on the surface of *P. falciparum*-infected erythrocytes [65]. KAHRP contains a 63-amino acid histidine-rich region, two variable repeat tandem regions, and several protein-binding domains that facilitate the interaction with different parasite and host proteins involved in a cytoadhesion complex [62,66]. Disruption of genes involved in PfEMP1 trafficking and the *kahrp* gene cause dramatic reduction in the formation of knobs and cytoadherence, which may be equivalent to the ridges present on the surface of *B. bovis* infected erythrocytes [65,67]. Additionally, down-regulation of chaperone proteins involved in PfEMP1 transport may affect *var* gene regulation in severe malaria [68]. Like KAHRP, *sbp2t11* has an N-terminal signal peptide and a PEXEL-like motif responsible for its maturation and trafficking to spherical bodies. Some proteins staged within the spherical bodies, such as the Smorf proteins, undergo release during or immediately following erythrocytes invasion. SBP2t11 may be involved in staging of proteins, or may directly influence cytoadhesion. Data so far collected from *Babesia* infected erythrocytes do not distinguish between these possibilities. No significant sequence homology exists between *Plasmodium* and *Babesia* proteins known to be involved in cytoadhesion, and it is likely that these proteins have evolved independently because of convergent evolution, perhaps playing similar functional roles in these two distinct parasites. 

## 7. Concluding Remarks: Proposed Model of a Possible Mechanism of Attenuation in *Babesia bovis*

Based on experimental data, it is reasonable to speculate that the *B. bovis* VESA and SBP2 proteins have similar characteristics and roles as PfEMP1 and KAHRP *in P. falciparum*, respectively. VESA proteins have been involved in cytoadhesion and have undergone antigenic variation encoded by the *ves* multigene family. The VESA proteins are responsible for the adherence to unknown host receptors and, like the *Plasmodium* PfEMP proteins, are deemed as *B. bovis* virulence factors (Figure 4). Yet, although no significant sequence similarities exist between the PfEMP and VESA proteins, they both seem to share similar features and functional roles. The intracellular C-terminal portion of VESA could also be interacting with the parasite proteins involved in the formation of ridges and cytoskeleton host proteins (Figure 4) as is the case with PfEMP and KAHRP in *Plasmodium* [62,63]. Similar to KAHRP, the SBP2 proteins contain an N-terminal signal peptide, PEXEL motif, form tandem repeat regions (just in SBP2) and protein binding domains.

Based on recent experimental evidence and similar findings in related parasites, we propose a model of modulation of virulence based on the interplay of parasite proteins that are secreted into the erythrocyte cytoplasm and on the surface of the infected erythrocyte. We based our model on the known VESA proteins, the only so far well-characterized virulence factor in *B. bovis* and the SBP2t11 protein, the first attenuation factor described, but no doubt, other still uncharacterized proteins may play important roles in this process (Figure 4). This model suggests that SBP2 truncated proteins, such as SBP2t11, might be involved in protein-protein interactions in a mode that inhibits or regulates ridge formation in *B. bovis* infected erythrocytes. Thus, overexpression of the truncated SBP2t11 may tip the balance on the attenuation side by decreasing the number of ridges in infected cells, concomitant with reduced cytoadhesion and parasite virulence (Figure 4). This model is supported by the structural features shared among *sbp2t11* and similar exported PEXEL processed *Plasmodium* proteins involved in knob formation, which is the confirmed observations that SBP2 truncated protein, especially SBP2t11, is over-expressed in attenuated phenotypes, and the findings of the current study demonstrate that infected erythrocytes overexpressing SBP2t11 have a reduced cytoadhesion phenotype [53]. In summary, current data are consistent with the hypothesis that over-expression of SBP2t11 interposes with the protein network described for the virulent phenotype, reducing ridge formation, and cytoadherence in attenuated parasite, suggesting SBP2t11 as a possible modulator of virulence in *B. bovis* (Figure 4). More research to identify novel attenuation and virulence factors in *B. bovis* is therefore needed to clarify this process further, filling a research gap that can be exploited for generating new vaccines and methods for the control of bovine babesiosis.
